# A novel 1-bp deletion in *PITX3* causing congenital posterior polar cataract

**Published:** 2011-05-06

**Authors:** Vanita Berry, Peter J. Francis, Quincy Prescott, Naushin H. Waseem, Anthony T. Moore, Shomi S. Bhattacharya

**Affiliations:** 1 Department of Genetics, Institute of Ophthalmology, University College London, London, UK; 2 Casey Eye Institute, Oregon Health and Science University, Portland, OR; 3 Moorfields Eye Hospital, London, UK

## Abstract

**Purpose:**

Cataracts are the most common cause of blindness worldwide. Inherited cataract is a clinically and genetically heterogeneous disease. Here we report a novel mutation in the paired-like homeodomain 3 (*PITX3*) gene segregating in a four generation English family with an isolated autosomal dominant posterior polar cataract.

**Methods:**

A genome-wide linkage was performed by means of single nucleotide polymorphism (SNP) and microsatellite markers. Linkage analyses were performed with the GeneHunter and MLINK programs. Direct sequencing of PCR products was performed to detect mutation in the gene, using the BigDye version 3.1 and analyzed using Sequence analysis version 5.2.

**Results:**

Genome-wide linkage analysis with SNP markers, identified a disease-haplotype interval on chromosome 10q. Two point positive logarithm of odds (LOD) scores was obtained with markers D10S205 (Z=3.10 at θ=0.00), flanked by markers D10S1709 and D10S543, which harbors the homeobox gene *PITX3*. Sequence analysis of *PITX3* revealed a 1-bp deletion that cosegregated with all the affected members of this family which resulted in a frameshift in codon 181 and likely to produce an aberrant protein consisting of 127 additional residues.

**Conclusions:**

The 542delC is a novel mutation in *PITX3* causing an isolated posterior polar cataract.

## Introduction

Bilateral congenital cataract is the most common cause of treatable childhood blindness. Cataracts are phenotypically and genotypically heterogeneous, most show autosomal dominant inheritance with complete penetrance [1,2]. Less frequently, autosomal recessive and X-linked inheritance patterns are seen. There has been significant progress in identifying the molecular genetic basis of human cataract. Many genes have been implicated including those encoding the transparent intracellular lens proteins (crystallins), membrane gap junction proteins (connexins), water channel proteins (aquaporins), solute carrier protein (*SLC16A12*) various cytoskeletal proteins (e.g., phakinin, filensin, vimentin), transmembrane proteins (transmembrane protein 114 [*TMEM114*], lens intrinsic membrane protein [*LIM2*], chromatin modifying protein-4B [*CHMP-4B*], and EPH receptor A2 [*EPHA2*]) and transcription factors [3].

Transcription factors play an important role in the embryological development of the lens including the interaction between the embryonic surface ectoderm and the budding optic vesicle. This interaction is critical for normal lens induction [4]. Mutations in several transcription factor genes notably paired box 6 (*PAX6*)*, *forkhead box E3 (*FOXE3*), eyes absent homolog 1 (Drosophila; *EYA1*), and v-maf musculoaponeurotic fibrosarcoma oncogene homolog (avian; *MAF*), and paired-like homeodomain 3 (*PITX3*) have been implicated in both congenital cataract and anterior segment mesenchymal dysgenesis (ASMD) [5-10]. Here we report a novel 1-bp deletion (542delC) mutation in *PITX3* in a family with an isolated autosomal dominant posterior polar cataract.

## Methods

### Phenotyping

The family in this study was identified through the proband attending the cataract clinic at Moorfields Eye Hospital, London, UK. The local ethics committee approval was obtained for the studies and all individuals taking part in the study gave written informed consent. Both affected and unaffected family members underwent full ophthalmic examination, with careful slit lamp examination. In this pedigree all the affected individuals were diagnosed as having isolated posterior polar cataract.

### Genotyping and linkage analysis

Genomic DNA was extracted from EDTA-sequestered blood samples using the Nucleon II DNA extraction kit (Scotlab Bioscience, Strathclyde, Scotland, UK).

Genotype data of the individual family members were generated using the GeneChip Human Mapping 50K Array Xba 240 and Assay Kit from GeneChip Human Mapping 100k Set (Affymetrix, High Wycombe, UK). Initial checks of the results were performed with GeneChip Command Console Viewer (v1.1.0.845). Genotyping Console (v3.0.2) assigned individuals’ genotypes. Alohomora version 0.30 (Max Delbrück Center for Molecular Medicine, Berlin, Germany) was used to prepare the raw genotype data for linkage analysis and for PedCheck (version1.1, Jeff O’Connell; University of Pittsburgh, Pittsburgh, PA.) to detect and remove Mendelian Errors (ME) from the data. Genehunter (version 2.1_r5 beta) was used to perform the subsequent parametric linkage analysis with dominant inheritance and full penetrance, of the disease allele with a frequency of 0.0001 in the general population.

The region showing significant logarithm of odds (LOD) score was refined using markers from Marshfield, GDB Human genome database and Ensemble databases. Analysis was performed using GeneMapper (version 4.0, Applied biosystems, Warrington, UK) on a ABI PRISM 3730 Genetic Analyzer (Applied Biosystems, Warrington, UK). A full penetrance and a gene frequency of 0.0001 were used for the cataract locus. Two-point linkage analysis was performed using the MLINK component of the LINKAGE program package version 5.10. The pedigree and haplotype data was managed by Cyrillic software (version 2.1.3).

### Sequence analysis

Genomic DNA from all the individuals was amplified using PCR Reddy Mix (AB gene; Thermo Scientific, Epsom, UK) and *PITX3*-specific primers (Table 1). Samples were processed through 30 cycles of amplification consisting of 30 s at 94 °C, 30 s at 60 °C, and 45 s at 72 °C. The final step was lengthened to 5 min. Direct sequencing of PCR products was performed using the BigDye version 3.1 (Applied Biosystems) on a ABI 3730 DNA Analyzer and analyzed using Sequence analysis version 5.2.

**Table 1:** Primers for *PITX3*.

**Set**	**Forward primer**	**Reverse primer**	**Annealing temp (°C)**	**Product size (bp)**
Exon 1	ccggctgggggtggcagtacgcgg	ggtccagcaatagctcctcggccc	60	237
Exon 2	cagctttacggctggggttgag	ggatgaagctgttatgtcctgcac	60	296
Exon 3	gggagccagcgagtggcttaggag	gggtggaaccgctggcctccg	60	374
Exon 4	gtctctagccacctcatctc	ctggggcgggagcaagccagtc	60	808

## Results

**Figure 1:**
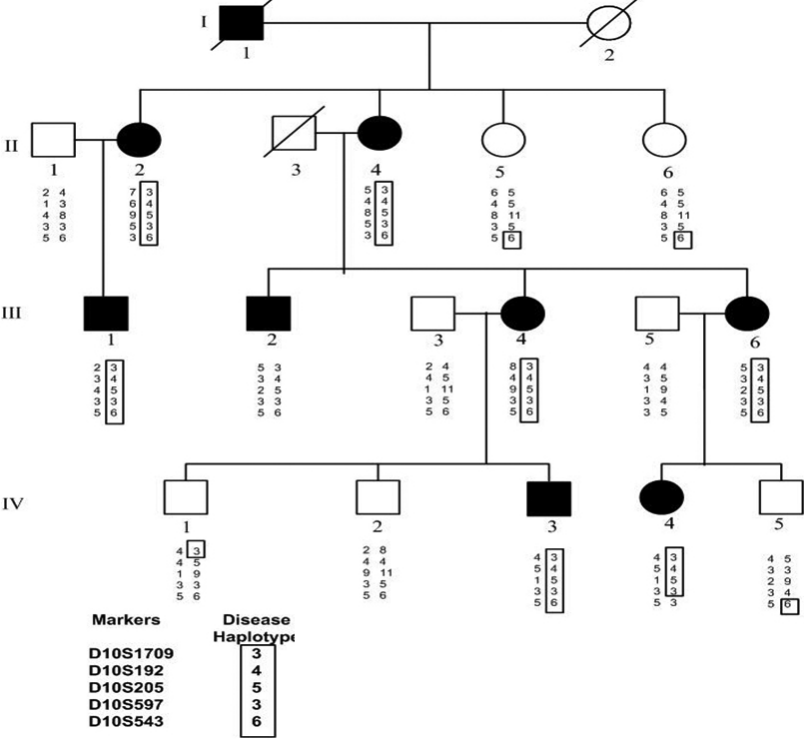
Abridged pedigree of the posterior polar cataract family used in this study showing the segregation of five chromosome 10q markers listed in descending order. Squares and circles symbolize males and females respectively. Open and filled symbols indicate unaffected and affected individuals. The disease haplotype is shown in the box.

**Figure 2:**
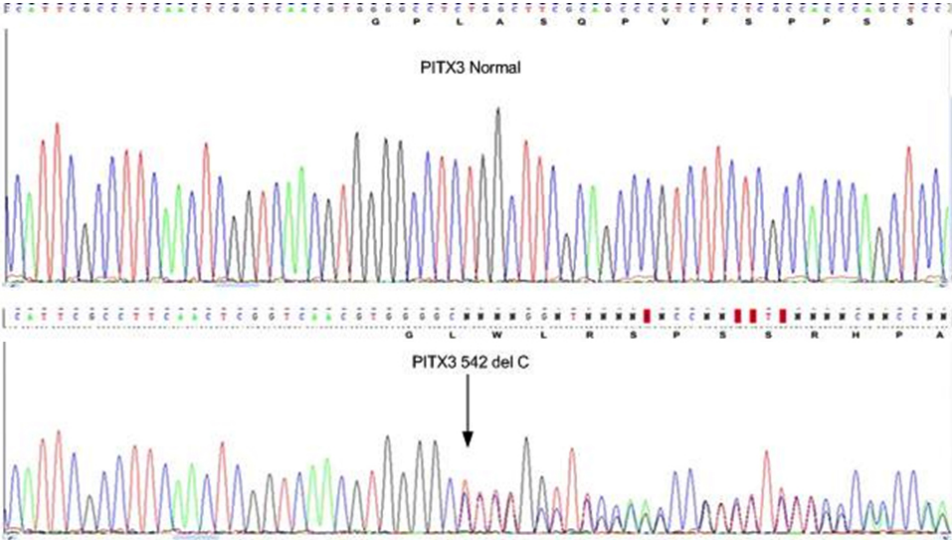
Sequence analysis of *PITX3* with normal and 1-bp deletion fragment showing a frame shift in an affected individual.

A four-generation family with posterior polar cataract, comprising 16 members of the pedigree (Figure 1) including 8 affected individuals, 5 unaffected individuals, and 3 spouses were genotyped with SNP markers using GeneChip Human Mapping 50K Array. Linkage analysis identified a likely disease-haplotype interval on chromosome 10q (rs911579-8Mb-rs2286396). Further, microsatellite markers were used to narrow down the region on chromosome 10q25. Two point positive LOD score was obtained with markers D10S205 (Z=3.10 at θ=0.00), flanked by markers D10S1709 and D10S543 (Table 2). This area encompasses the homeobox gene *PITX3* between markers D10S192 and D10S205. *PITX3* comprises four exons and encodes a protein of 302 amino acid residues. Sequence analysis of this gene revealed, in exon 4, a 1-bp deletion (542delC; Figure 2) that cosegregated with all the affected members of PPC family. It resulted in a frameshift in codon 181 and likely produced an aberrant protein consisting of 127 additional residues. This mutation found in *PITX3* affected the region outside the homeodomain in the COOH-terminal end of the protein and result primarily in posterior polar cataract. This change was not seen in 200 healthy individuals.

**Table 2:** Two-Point LOD scores for linkage between the *PITX3* locus and 10q25 markers.

** **	**Distance**	**Z at θ=**						
Marker	cM	0.0	0.01	0.05	0.1	0.2	0.3	0.4
D10S1709	4.52	1.80	1.79	1.71	1.55	1.14	0.64	0.20
D10S192	1.19	0.52	0.51	0.45	0.40	0.30	0.24	0.14
D10S205	3.33	3.10	3.05	2.83	2.53	1.90	1.21	0.52
D10S597	0.00	0.82	0.81	0.74	0.65	0.47	0.27	0.09
D10S543		−4.44	−1.52	−0.79	−0.47	−0.20	−0.10	−0.07

## Discussion

Posterior polar cataract (PPC) is a clinically distinct opacity that is located at the back of the lens and, because of its proximity to the optical center of the eye, can have a marked effect on visual acuity. Previously, PPCs have been described in association with mutation in five genes (*EPHA2* on 1p36, *CRYAB* on 11q22-q22.3, *CHMP4B* on chromosome 20p12, *CRYBA1/A3* on 17q12, and *PITX3* on 10q25) [11-14]. *PITX3* encodes a paired-like class of homeobox transcription factor, a member of the *PITX* family, which also includes *PITX1* and *PITX2*. *PITX2* and *PITX3* are involved in eye development and are expressed in cornea, lens, and retina [15]. Mutations in *PITX2* have been linked to Rieger syndrome causing glaucoma and mild craniofacial dysmorphism in humans [16]. In the aphakia mouse mutant, two deletions in the promoter of the homeobox transcription factor *Pitx3* lead to loss of its function and to arrest of eye development at the lens stalk stage [17]. Mutations in the homologus human *PITX3* gene have been demonstrated to cause cataracts and anterior segment dysgenesis.

So far three different mutations in *PITX3* have been reported in man. The first mutation was a COOH-terminal 17-bp insertion (657ins17) that resulted in a frame shift and abnormal configuration of nearly one third of the protein. This mutation was found in a large family with anterior segment ocular dysgenesis and cortical cataracts [9]. Several recent studies have shown a recurrence of the same 17-bp insertion mutation in number of families of different ethnic backgrounds affected with congenital posterior polar cataract that, in some cases, included anterior segment defects [10]. The second mutation was a serine to asparagine substitution in the NH_2_-terminal region of the protein (S13N) [9]. An additional COOH-terminal single-nucleotide deletion, 650delG was identified in two families affected with posterior polar cataract; this mutation is predicted to result in a truncation of the normal protein around the same site two amino acids upstream as the recurrent 17-bp insertion [10,18]. Homozygous mutation for 650delG has been found in two siblings from consanguineous marriage causing microphthalmia and central nervous system defects [18].

The PITX3 protein mutantions S13N and G219fs have been shown to alter the DNA-binding profiles and transactivation activities and there is a partial loss-of-function in both mutants with the G219fs form being more severely affected. The G219fs mutation was found in multiple families affected with congenital cataracts along with anterior segment malformations in many members. These findings suggested that the presence/severity of anterior segment defects in families affected with G219fs may be determined by secondary factors that are expressed in the developing anterior segment structures and may modify the effect(s) of this mutation [19]. *PITX3* is expressed in the developing lens, skeletal muscle, and dopaminergic neurons of the substantia nigra in the brain. Recently *PITX3* polymorphisms have been shown to be associated with Parkinson disease [20].

Here we report a novel mutation (542delC) in *PITX3* causing an isolated posterior polar cataract in an English pedigree. This 1-bp deletion mutation in exon 4 of *PITX3,* resulted in a frameshift in codon 181 that may lead to the production of an aberrant protein consisting of 127 additional residues at the COOH-terminal region. This region is thought to be involved in complex protein–protein interactions, imparting specificity and efficiency to homeoprotein function [19]. This mutation does not affect the homeodomain region of the protein but highlights the significance of the COOH-terminal region, which already have been associated with the disease.
